# Safety and efficacy of short-term oral immunotherapy with Cry j 1-galactomannan conjugate for Japanese cedar pollinosis: a randomized controlled trial

**DOI:** 10.1038/srep46142

**Published:** 2017-04-11

**Authors:** Daisuke Murakami, Motohiro Sawatsubashi, Hirofumi Omori, Akira Saito, Akio Kato, Shizuo Komune, Takashi Nakagawa

**Affiliations:** 1 Department of Otorhinolaryngology, Graduate School of Medical Sciences, Kyushu University, Fukuoka, Japan; 2 Department of Otorhinolaryngology, Saiseikai Fukuoka General Hospital, Fukuoka, Japan; 3 Bio & Healthcare Business Group, Bio & Healthcare Business Division, Wako Filter Technology Co., Ltd., Ibaraki, Japan; 4 Department of Biological Chemistry, Yamaguchi University, Yamaguchi, Japan

## Abstract

Current allergen-specific immunotherapy (AIT) for pollinosis requires long-term treatment with potentially severe side effects. Therefore, development of an AIT that is safe and more convenient with a shorter regimen is needed. This prospective, double-blind, placebo-controlled trial randomized 55 participants with Japanese cedar pollinosis (JCP) to active or placebo groups to test the safety and efficacy of short-term oral immunotherapy (OIT) with Cry j 1-galactomannan conjugate for JCP. Mean symptom-medication score as the primary outcome in the active group improved 27.8% relative to the placebo group during the entire pollen season. As the secondary outcomes, mean medication score in active group improved significantly, by 56.2%, compared with placebo during the entire pollen season. Mean total symptom score was similar between active and placebo groups during the entire pollen season. There were no severe treatment-emergent adverse events in the active and placebo groups. Therefore short-term OIT with Cry j 1-galactomannan conjugate is safe, and effective for reducing the amount of medication use for JCP.

The prevalence of Japanese cedar pollinosis (JCP) has been increasing, and is currently around 30%^1^,^2^. About 70% of patients with JCP are also allergic to Japanese cypress pollens, which are highly homologous with Japanese cedar (JC) pollen allergens^3^.

Allergen-specific immunotherapy (AIT) can alter allergic disease, induce immune tolerance, and represents a potentially curative treatment^4^,^5^. In current clinical practice, subcutaneous immunotherapy (SCIT) or sublingual immunotherapy (SLIT) is available. However, SCIT in Japan is not widespread because of the need for frequent medical visits over 3–5 years, injection pain, and potential severe side effects, including anaphylaxis. Recently, SLIT for JCP was approved by the Japanese Ministry of Health, Labour and Welfare as a safe form of immunotherapy compared with SCIT^6^,^7^. However, SLIT requires years of treatment before the therapeutic effect is apparent^8^ and the effect may be weaker than SCIT^6^. In addition, real-life persistence is better in SCIT users than in SLIT users^9^, although it is low overall. Therefore, development of an AIT that is safe, effective and more convenient with a shorter regimen is needed.

Although there has been controversy about the efficacy of oral immunotherapy (OIT) for pollinosis, OIT has been used successfully to treat food allergy^10^,^11^,^12^. OIT for pollinosis has advantages compared with SCIT/SLIT. Subjects can ingest larger amounts of antigen each time and OIT is expected to induce immune tolerance over a short period because a large number of immune cells are present in the intestinal tract^13^. Although no severe systemic side effects were reported, OIT induced many gastrointestinal adverse effects (AEs) because the allergens were given in their native form^14^. Therefore, gastrointestinal AEs and structural failure of the antigen due to digestive enzymes in the stomach should be avoided. That is a key point whether OIT for pollinosis is successful.

Cry j 1 is a major allergen of JC pollen^15^. In previous studies, we reported that when Cry j 1 is conjugated to galactomannan, its IgE-binding epitopes are masked^16^. Furthermore, this conjugate resists degradation by digestive enzymes and traffics efficiently to dendritic cells in the gut lumen^17^. In addition, our recent open-label study without placebo has shown that short-term OIT with Cry j 1-galactomannan conjugate for JCP was safe, effective and induced antigen-specific immune responses^18^. Therefore further study is required for the confirmation of efficacy and safety. Here, we report a prospective, randomized, double-blind, placebo-controlled trial for safety and efficacy of short-term OIT with Cry j 1-galactomannan conjugate for JCP.

## Methods

### Participants

**Figure 1:**
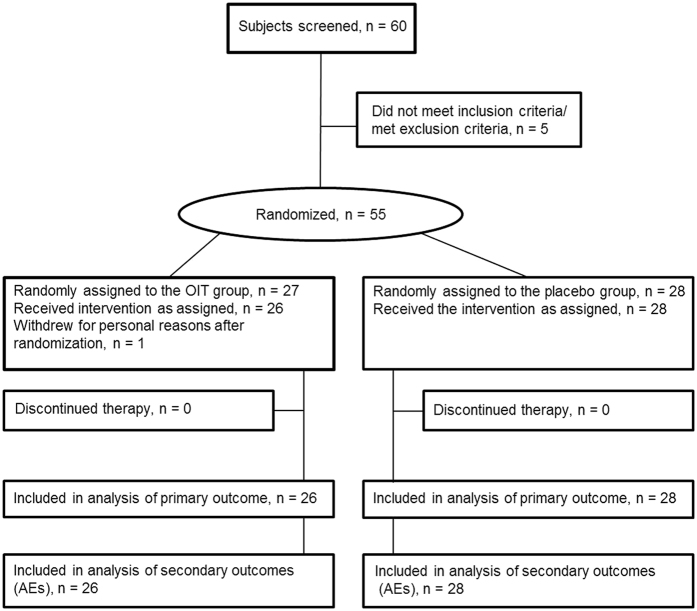
Study flow chart. Number of individuals assessed for the trial. AE, adverse events.

Sixty patients were screened for this study (Fig. 1). 55 adult Japanese participants (29 male, age range 22–65) were recruited. All have moderate or severe rhinoconjunctivitis caused by JC pollens and were otherwise healthy. They had received pharmacological treatment for the last three consecutive cedar pollen seasons, and lived in or around Fukuoka City, Japan, where a similar amount of pollen spread was expected. The diagnosis of JCP was based on clinical history and serum cedar-specific IgE levels associated with a score of 2 or greater using ImmunoCAP System (Thermo Fisher Scientific/Phadia, Uppsala, Sweden). Exclusion criteria were as follows: severe asthma, chronic sinusitis, previous immunotherapy or ongoing immunotherapy with other allergens, treatment with β-blockers or those on continuous corticosteroids, pregnancy or planned pregnancy, participation in another clinical trial, and other standard contraindications for immunotherapy^19^. Informed consent was obtained from all participants. The study was conducted according to the principles in the Declaration of Helsinki, and was approved by the Institutional Ethics Committee of KyushuUniversity Hospitals (number 24102), Saiseikai Fukuoka General Hospital (number 2013–28) and registered in UMIN-CTR (UMIN000011995) on 9 October 2013.

### Study design

We performed a prospective, randomized, double-blind, placebo-controlled trial with 2 parallel arms between 6 January 2014, approximately 6 weeks before the expected start of the pollen season, and 21 May 2014, after the pollen season. The sample size was estimated based on our recent open studies^18^,^20^. Participants were recruited between 17 October 2013, and 31 December 2013 and screened for eligibility by physicians at Kyushu University Hospitals and Saiseikai Fukuoka General Hospital in Fukuoka City, Japan. Randomization was performed by an independent survey research firm (IBERICA Co., Ltd., Fukuoka, Japan). The allocation officer of the independent survey research firm made a randomization code and assigned a number to the study drug based on the randomization code. The random allocation sequence was generated using the permuted block method (block size 2) and stratification by sex and per center. First, a physician sent the information about the screened participant to the allocation officer from the independent survey research firm through an independent data center by fax. Second, the allocation officer received that information and sent an assigned drug number to the physician through the same independent data center by fax. Finally, the physician prescribed the study drug of the assigned number to the participant.

Participants were randomized 1:1 to 2 groups; one group received daily OIT with Cry j1 extract allergen-galactomannan conjugate in a capsule formulation according to the OIT regimen, and the other group received placebo, which matched the active treatment in size, shape, and color but contained no pollen allergens or other active ingredients. Twenty-seven subjects were randomly assigned to the OIT group. One man in the OIT group withdrew for personal reasons before commencement of OIT. The active OIT group consisted of 26 Japanese participants (14 male, age range 22–65). The placebo group consisted of 28 Japanese participants (14 male, age range 23–60). Physicians and participants were blinded to the assigned intervention groups until the analysis of outcomes was completed at the end of the study.

### Cry j 1-galactomannan conjugates

Standardized JC pollen antigen-galactomannan conjugates were manufactured by Wako Filter Technology Co., Ltd. (Ibaraki, Japan) and were of Good Manufacturing Practice grade^16^. A capsule of JCP antigen-galactomannan conjugate contained 187.5 μg of Cry j 1.

### OIT and pharmacological treatment

**Figure 2:**
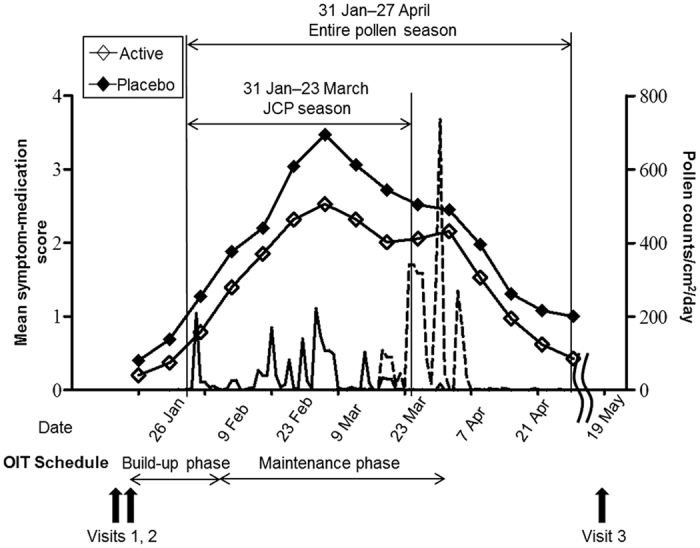
Study design, symptom-medication score and daily Japanese cedar/cypress pollen counts. Solid line: Japanese cedar pollen counts. Broken line: Japanese cypress pollen counts. Open squares: symptom-medication score in the active group (n = 26). Solid squares: symptom-medication score in the placebo group (n = 28). Statistical analysis was performed using unpaired t-test between active and placebo groups. Visit 1: early January 2014, screening of potentially eligible subjects. Visit 2: mid-January 2014, the beginning of the build-up phase of OIT after randomization. Visit 3: middle of May 2014, after the entire pollen season.

In the build-up phase, the dose was gradually increased to a maintenance dose over 18 days from the middle of January 2014. For the first 6 days, one capsule was given orally after breakfast; for the next 6 days, one capsule was given twice daily (after breakfast and the evening meal); and for the subsequent 6 days, three capsules were given (two after breakfast and one after the evening meal). Thereafter, two capsules were given twice daily during the maintenance phase for 51 days from the beginning of February 2014 to the end of March 2014. Participants in the control group received placebo capsules as per the active OIT group. Physicians (from the Nasal Allergy Study Group of the Department of Otorhinolaryngology, Graduate School of Medical Sciences, Kyushu University) examined the participants of both groups in that two hospitals, and checked blood samples before the JCP season and OIT (visits 1, 2), and after the pollen season (visit 3) in both groups (Fig. 2). In addition to OIT, the participants received pharmacological treatment for rhinoconjunctivitis caused by JCP throughout the pollen season according to the Japanese Guidelines for Allergic Rhinitis^21^. Participants carefully recorded the mean nasal and ocular symptom score and usage of rescue drugs (antihistamines, etc) per week in their pollen electronic file diaries which were sent to the web site of an independent survey research firm during the pollen season. Data were managed, collected, and analyzed by the independent survey research firm at study end.

### Pollen counts

The mean annual amount of cedar and cypress pollen in Fukuoka was measured using Durham pollen samplers in two different areas: Fukuoka City Medical Association Hospital and Fukuoka National Hospital.

### Adverse events

Treatment-emergent adverse events (TEAEs) were monitored throughout OIT and were graded according to the Common Terminology Criteria for Adverse Event (CTCAE) v4.03/MedDRA v12.0. Briefly, AEs were graded as mild (grade 1), moderate (grade 2), severe (grade 3), life-threatening (grade 4), or death (grade 5). Discontinuation criteria for OIT were grade ≥ 3 AEs or grade ≤ 2 AEs if the participant wished to withdraw from the study. The occurrence of TEAEs with OIT was assessed as a secondary outcome.

### Symptoms and medication use

During the cedar/cypress pollen season, participants recorded their weekly symptoms of rhinoconjunctivitis evaluated on a scale of 0–4 in accordance with the Japanese Allergic Rhinitis QOL Standard Questionnaire No. 1 (JRQLQ No 1)^22^. Total symptom score was the sum of each component score: none, 0; mild, 1; moderate, 2; severe, 3; and very severe, 4. Nasal and ocular symptoms covered by the questionnaire included runny nose, sneezing, nasal congestion, itchy nose, itchy eyes and watery eyes. Total medication score each week during the cedar/cypress pollen season was also calculated and recorded according to drug type and duration of usage, based on the Practical Guideline for the Management of Allergic Rhinitis in Japan^21^,^22^ as follows: antihistamines and topical ocular antihistamines – 1; topical nasal steroid sprays and ocular steroid drops – 2; and oral corticosteroids – 3. Weekly total medication score was converted to a daily medication score in that week. Thus medication scores represented the mean daily medication score in 1 week.

First, participants were treated with antihistamines (Levocetirizine hydrochloride, Fexofenadine hydrochloride) as rescue medications for symptom relief. If symptoms were not improved and participants desired more drugs, we prescribed nasal steroid sprays (Mometasone furoate, Fluticasone furoate) and/or ocular antihistamine drops (Ketotifen fumarate, Olopatadine hydrochloride) according to symptoms in addition to the initial rescue medication. If symptoms were not improved and participants desired more drugs, we prescribed topical ocular steroid drops (Fluorometholone). Oral corticosteroid use was limited unless participants could not endure symptoms using other anti-allergic medications. Other anti-allergic medications except for oral corticosteroids were used in their conventional amounts without particular limitation according to JCP symptoms. Symptom-medication scores reflected the mean total symptom score (total symptom score/6, with 4 points for the maximum value) plus the mean daily medication score in 1 week.

### Study outcomes

The primary outcome was efficacy of OIT with the Cry j 1-galactomannan conjugate for JCP assessed by examining the mean symptom-medication score during the Japanese cedar/cypress (entire pollen) season. The secondary outcome was efficacy of OIT with the Cry j 1-galactomannan conjugate for JCP assessed by examining the mean total symptom score and medication score during the entire pollen season. An additional secondary outcome was safety of OIT with the Cry j 1-galactomannan conjugate for JCP assessed by TEAEs monitored throughout OIT.

### Population and statistical analysis

The safety population included all participants receiving at least one dose of the study medication. The ITT population included all randomly assigned participants receiving at least one dose of the study medication, who recorded their weekly symptoms of rhinoconjunctivitis, usage medication, and AEs with OIT on the survey research firm web site at least once. Primary and secondary efficacy variables were analyzed by analysis of covariance (ANCOVA), with symptom-medication score in the pre-pollen season, and presence or absence of sensitization of cypress pollen and house dust mites. Statistical analyses, including Fisher’s exact probability test, unpaired *t*-test, and ANCOVA were performed using SAS 9.3 (SAS Institute Inc., Cary, NC, USA). Differences were considered statistically significant when *p* < 0.05. All statistical calculations except for AEs were undertaken by an independent survey research firm (IBERICA Co., Ltd., Fukuoka, Japan).

## Results

### Participant flow and characteristics

Figure 1 shows the overall participant study flow. No participants undergoing active treatment or undergoing placebo treatment withdrew from the study during OIT and the entire pollen season. In the ITT analysis of primary outcome and AEs, 26 participants in the active OIT group and 28 participants in the placebo group fully complied with the study protocol. Characteristics of each group are presented in Table 1. No differences in baseline characteristics were observed.

### Pollen counts

In 2014, the JCP season started in late January and ended in late March in Fukuoka City (Fig. 2). Although the start of the OIT regimen in this study was scheduled from 1 month before the JCP season, OIT started about 2 weeks before the JCP season because cedar pollen began to disperse faster than usual. The mean annual amount of cedar pollen was 1936/cm^2^ in Fukuoka. The Japanese cypress pollen season started in mid-March and ended in late April (Fig. 2). The mean annual amount of cypress pollen was 3342/cm^2^ in Fukuoka.

### Adverse effects

A total of 9 episodes of TEAEs were reported by 6 (23.0%) of 26 participants in the active group, while 5 (17.8%) of 28 participants in the placebo group reported a total of 7 TEAEs (Tables 2 and 3). All TEAEs were mild (grade 1) in severity for both treatment groups. There was no statistically significant difference between the treatment groups with respect to TEAEs rate in treatment, build-up, and maintenance phases (*p* = 0.444, 0.450, and 0.665, respectively). During the build-up phase, 5 of 26 (19.2%) participants in the active group had grade 1 TEAEs. These AEs comprised abdominal pain (n = 3), diarrhea (n = 1), stomach pain (n = 1), bloating (n = 1), and rhinorrhea (n = 1). Of the 5 participants with TEAEs, 4 had gastrointestinal AEs within 1 week after initiation of OIT. Diarrhea occurred in 1 patient when the dose increased to 2 capsules per day. The next day, the dose was reduced to one capsule per day and increased gradually as scheduled in the regimen. The diarrhea was resolved in 1 day without treatment and OIT was continued to the study end. In other participants with gastrointestinal AEs, the symptoms resolved within a few days or 26 days without any change of regimen or treatment (Table 3). One participant had rhinorrhea immediately after OIT, but this resolved after 1 day without treatment.

In the placebo group, 4 of 28 (14.2%) participants had grade 1 TEAEs, namely oral dysesthesia (n = 1), chest discomfort (n = 1), laryngopharyngeal dysesthesia (n = 1), loose stools (n = 1), and watering eyes (n = 1). Two of 4 participants with TEAEs had gastrointestinal AEs described above. One had oral dysesthesia 13 days after OIT, but this resolved after 1 week without treatment. Another had loose stools 17 days after OIT, which continued for 46 days and was resolved without treatment. Two participants had chest discomfort, laryngopharyngeal dysesthesia, or watering eyes immediately after OIT, but these resolved after a few hours or 1 day without treatment (Table 3).

During the maintenance phase, 2 of 26 (7.6%) participants in the active group had grade 1 gastrointestinal TEAEs (Tables 2 and 3). Two participants had stomach pain at 41 or 25 days after OIT, but symptoms resolved after 1 or 2 days without treatment, respectively (Table 3). In the placebo group, 2 of 28 (7.1%) participants had grade 1 gastrointestinal TEAEs. Two participants had abdominal pain at 34 or 19 days after OIT, but symptoms resolved after 6 or 3 days without treatment, respectively (Tables 2 and 3).

### Clinical efficacy

The mean symptom-medication scores and pollen counts in the community during the Japanese cedar/cypress (entire pollen) season are shown in Fig. 2. Temporal profiles of mean symptom-medication scores were lower in the active group compared with placebo from 2 February to 28 April 2014, during the entire pollen season, but were not statistically significant. Temporal profiles of mean total symptom scores in the active and placebo groups were comparable during the entire pollen season (Supplementary Fig. 1a). In contrast, the temporal profiles of the mean medication scores were lower in the active group compared with those in the placebo group during the entire pollen season, and they were significantly different on 23 February and on 2, 9, and 16 March 2014 (Supplementary Fig. 1b) (*p* = 0.028, 0.028, 0.017, and 0.036, respectively).

**Figure 3:**
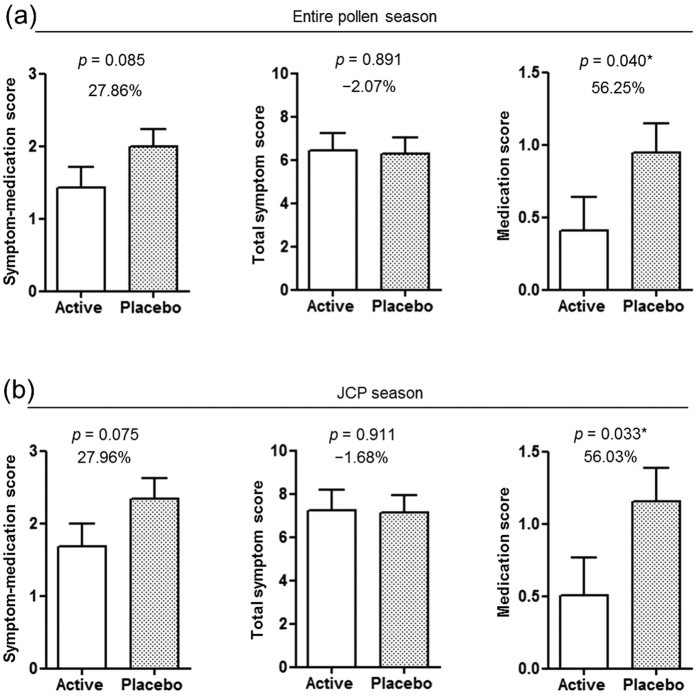
Percentage reduction in symptom-medication score, total symptom score, and medication score. (**a**) Japanese cedar/cypress pollen season (entire pollen season). (**b**) Japanese cedar pollen (JCP) season. Open squares: active group (n* = *26); solid square: placebo group (n* = *28). Data are least square (LS) mean weekly score ± SEs during entire pollen or JCP season. *p < 0.05, between active and placebo groups. Statistical analysis was performed using ANCOVA.

The primary outcome (mean symptom-medication score) improved relative to placebo in the active group compared with placebo during the entire pollen season and JCP season was but was not statistically significant (Table 4, Fig. 3a,b) (27.8% and 27.9% relative to placebo, *p* = 0.085, and 0.075, respectively). Among the secondary outcomes, mean total symptom score was the same in both groups during the entire pollen season and JCP was not statistically different (Table 4, Fig. 3a,b) (−2.0% and −1.6% improvement relative to placebo, *p = *0.891, and 0.911, respectively). In addition, mean total nasal score, total ocular score, and each symptom (sneezing, runny nose, nasal congestion, itchy nose, itchy eyes and watery eyes) score was not statistically different between both groups during the entire pollen season and JCP season (Table 4, Supplementary Table 1). On the other hand, the mean medication score was significant lower in the active group compared with placebo during the entire pollen season and JCP season (Table 4, Fig. 3a,b) (56.2% and 56.0% improvement relative to placebo, *p* = 0.040, and 0.033, respectively). Only 1 participant in the active group received corticosteroids (Betamethasone 0.25 mg/d-Chlorpheniramine Maleate 2 mg) for 2 days during peak JCP season to relieve symptoms.

## Discussion

Although there has been controversy about the efficacy of OIT for pollinosis, OIT has been successfully used to treat food allergy^10^,^11^,^12^. Additionally, our recent open-label study lacking a placebo has shown that short-term OIT with Cry j 1-galactomannan conjugate is safe and effective for JCP and that it induced antigen-specific immune responses^18^. Here, we report a prospective, randomized, double-blind, placebo-controlled trial for the safety and efficacy of short-term OIT with Cry j 1-galactomannan conjugate for treating JCP.

The primary outcome, symptom-medication score (combined mean total symptom score and medication score) was used to assess OIT efficacy. The mean symptom-medication score in the active group trended lower than that in the placebo group and was improved by 27.8% and 27.9% relative to that in the placebo group during the entire pollen season and during the JCP season, respectively, but this difference did not reach statistical significance (Fig. 3a,b). Among the secondary outcomes, the mean total symptom score was similar in the two groups during both the entire pollen season and the JCP season. In contrast, the mean medication score was significantly lower in the active group compared with that in the placebo group during both the entire pollen season and the JCP season (Table 4, Fig. 3a,b). Thus, the trend in the primary outcome between the active and placebo groups was almost entirely due to the reduction in medication usage, as shown by the significant changes in the secondary outcome variable.

In this study, the mean symptom-medication score as the primary outcome was analyzed by ANCOVA as previously described^23^,^24^, with symptom-medication scores in the pre-pollen season and the presence or absence of sensitization. However, a disadvantage of this method is that the score at each time point in each patient is not included in the analysis of the primary outcome. Notably, when including the score at each time point in each patient in the ANCOVA, the mean symptom-medication score as a primary outcome was similar to the medication score, and it, but not the total symptom score, was significantly lower in the active group than that in the placebo group during both the entire pollen season and the JCP season (*p* < 0.001 and 0.001, respectively) (Supplementary Table 2).

SCIT/SLIT have shown efficacy in large-scale random clinical trials (RCTs)^6^,^7^. Although a direct comparison with our results is difficult, the reduction rate in the primary outcome of RCTs of SCIT/SLIT for pollinosis was 20–30%^25^,^26^. Therefore, our primary outcome results indicate short-term OIT with allergen-galactomannan conjugates is as effective as conventional SCIT/SLIT for pollinosis. In addition, in the standard SCIT/SLIT regimen for pollinosis, it is necessary to start immunotherapy more than 6 months before the pollen season and continue it for years before its effectiveness is determined^7^. The period of immunotherapy is so long that the real-life persistence is very low overall in SCIT/SLIT users^9^. In contrast, OIT in this study started from 2 weeks before the pollen season and continued for about 2 months until the late period of the pollen season, which is much shorter compared with the standard SCIT/SLIT regimen. This length of treatment is the same as that of conventional drug therapy for JCP. Thus, good compliance is expected for this OIT regimen compared with the standard SCIT/SLIT regimen.

There are several possible explanations beyond the symptom elements for why the primary outcome was not improved by OIT. It is possible that the pharmacological treatments improved the symptoms to the same extent in the placebo and active groups. The participants in this study had moderate-to-severe rhinoconjunctive symptoms throughout the JCP season. It is difficult for participants to endure rhino-conjunctive symptoms without any pharmacological treatments. Therefore participants received pharmacological treatment for rhino-conjunctivitis according to the Japanese Guidelines for Allergic Rhinitis^21^ without limitations except for oral corticosteroids in addition to OIT. Many RCTs have shown that antihistamines (Levocetirizine hydrochloride, Fexofenadine hydrochloride), nasal steroid sprays (Mometasone furoate, Fluticasone furoate), ocular antihistamine drops (Ketotifen fumarate, Olopatadine hydrochloride) and ocular steroid drops (Fluorometholone) used as rescue medications improve rhino-conjunctive symptoms for pollinosis^27^,^28^,^29^,^30^. Thus, the rhino-conjunctive symptoms in the placebo and active groups may have been suppressed to the same extent by rescue medications. If true, this would explain why only the medication use, but not the symptomatic treatment, showed a significant improvement after OIT.

Alternately, the pollen season may have begun earlier than we expected, making the start of the OIT too late to be effective. Notably, the period from the start of OIT to the beginning of pollen season was shorter than originally scheduled. In the protocol, we expected a period of 1 month to the beginning of pollen season from the start of OIT, but the start time of pollen scattering in 2014 was early by 2 weeks compared with the average annual start time. Our previous 2012 open-label study without a placebo reported that participants receiving OIT showed significant improvements in total symptom scores, medication scores, and symptom-medication scores throughout the pollen season compared with the control group^18^. Although the placebo effect might have been lost, cedar pollen in 2014 began scattering about 3 weeks earlier compared with 2012. In addition, the total amount of cedar/cypress pollen in 2014 was greater than in 2012. This early pollen scattering and large amount of pollen might obscure differences in total symptom scores. Generally, during the course of AIT, early desensitization, mast cell and basophil activity for degranulation decrease immediately after the first administration of allergens. Next, T cell tolerance, modulation of antibodies, generation of allergen-specific regulatory T cells and suppression of allergic-specific Th1 and Th2 cells follow with increasing allergen-specific IgG4 as a blocking antibody over a period of weeks to months^4^. Indeed, our recent studies show serum IL-10 and antigen specific IgG4 were increased 1 month after OIT start with Cry j 1-galactomannan conjugate^18^,^20^. Therefore, it may take at about 1 month to obtain a therapeutic effect from OIT.

Another possibility is that the participants may have had varying disease severities, and some may not have had a sufficiently severe disease for our symptom scoring system to detect the changes in their symptoms. The symptom scoring system used in this study was based on reports of subjective severity by each participant. Therefore, if there were great variations in each individual symptom sore during the pollen season, this small scale study might not be capable of detecting the differences in symptom sores between the placebo and OIT groups. In fact, although it was not included in the outcomes of this study, the simple questionnaire carried out at the end of the pollen season in this study concerning the participants’ overall symptoms during the entire pollen season were compared with their symptoms in the previous years. Results indicated that the overall symptoms during this pollen season in the active group were significantly improved compared with those in the placebo group (*p* < 0.05) (Supplementary Fig. 2). Thus, this result supports the contribution of these factors to our findings. The safety of OIT for pollen allergy has been confirmed in many trials, and no severe systemic side effects were reported. However, many minor side effects (not life-threatening) were observed because the native form of the allergen was administered for OIT^31^. These effects tended to increase with an increased dosage of allergen^14^. It is therefore desirable to develop a new agent to suppress gastrointestinal AEs associated with OIT. Our recent open studies have reported that short-term OIT with the Cry j 1-galactomannan conjugate for JCP was relatively safe, but no placebo arm was used^18^,^20^. Thus, in this study, we assessed the safety of OIT with the Cry j 1-galactomannan conjugate compared with placebo.

In active groups, only mild (grade 1) AEs were observed in the build-up and maintenance phases of OIT, and there was no significant difference in the rate of mild (grade 1) AEs between both groups. In the active group, all grade 1 TEAEs were resolved without treatment within days or weeks. In the treatment phase of OIT, no severe AEs occurred. Furthermore, no participants in either group withdrew during OIT or the entire pollen season. In previous studies of OIT for food allergy, AEs were more frequent with initial day dose escalation and in the build-up phase. The rates of AEs were highest during the early period of OIT^11^,^12^. This tendency in this study applied to not only the active group but also placebo. Although there was no significant difference in the AE rate between both groups in the build-up phase, the rate of AEs associated with abdominal symptoms within 1 week from OIT start was higher in the active group compared with placebo (4/26 participants in active groups versus 0/28 participants in placebo, *P = *0.047). Thus, initial mild AEs associated with abdominal symptoms may be considered characteristic AEs of OIT with Cry j 1-galactomannan conjugate.

Although it is difficult to directly compare SLIT with OIT using Cry j 1-galactomannan conjugate, OIT reduced local AEs compared with SLIT which induced 40–75% local AEs in early treatment^32^ and can be expected to have good compliance for short-term regimens. Therefore, OIT with allergen-galactomannan conjugate for pollinosis may provide an alternative to conventional SCIT/SLIT.

The results of previous open trials conducted with the same protocol using Cry j 1-galactomannan conjugate showed that the levels of serum Cry j 1-specific IgE and IgG4 during the early period of OIT were significantly increased in the OIT group but not in the control group^18^,^20^. Although the immunization method and target disease are different in these studies, serum antigen-specific IgE and IgG4 elevation have been observed during the early period of immunotherapy in allergen-specific immunotherapies, including OIT, SCIT, and SLIT^5^,^11^. These results show that an antigen-specific immune response is certainly occurring and that this response is useful for the effectiveness of OIT using Cry j 1-galactomannan. Furthermore, our previous study showed that, during OIT, the level of IL-10 production by peripheral blood mononuclear cells (PBMCs) was increased in the OIT group compared with that in the control group, but there were no differences in the percentage or number of naturally occurring regulatory T cells (FoxP3^+^CD25^+^CD4^+^ T cells) in PBMCs between these two groups during pollen season or during OIT^20^. Additional research is needed to investigate these IL-10-producing cells and the mechanism of immune tolerance.

One study limitation was the small cohort. Further large-scale studies are required to determine whether short-term OIT with antigen-galactomannan conjugate is a universally effective method for the treatment of airway allergy.

In summary, we report that short-term OIT with Cry j 1-galactomannan conjugate is safe and effective for reducing the amount of medication use for JCP. Our findings suggest that OIT with allergen-galactomannan conjugates may permit a shorter and thus more convenient immunotherapy regimen for pollinosis compared with SLIT/SCIT.

## Additional Information

**How to cite this article**: Murakami, D. *et al*. Safety and efficacy of short-term oral immunotherapy with Cry j 1-galactomannan conjugate for Japanese cedar pollinosis: a randomized controlled trial. *Sci. Rep.* **7**, 46142; doi: 10.1038/srep46142 (2017).

**Publisher's note:** Springer Nature remains neutral with regard to jurisdictional claims in published maps and institutional affiliations.

## Supplementary Material

Supplementary Information

## Figures and Tables

**Table 1:** Participant Demographics.

Group	Active group	Placebo group	*p* value
Sex (M/F)	12/14	14/14	0.792
Mean (SD) age	40.4 (11.4)	40.4 (9.4)	0.981
Range	22–65	23–60	
Total IgE (IU/ml [SD])	126 (131)	191 (200)	0.168
Range	10–517	10–943	
Cedar-Specific IgE (UA/ml [SD])	16.2 (14.5)	19.2 (18.2)	0.508
Range	0.79–56.80	1.54–72.60	
Cypress-Specific IgE (UA/ml [SD])	2.4 (3.5)	2.3 (3.2)	0.931
Range	0.12–15.0	0.10–14.6	
Other allergies, No. (%)			
Cypress pollen allergy	23 (88.4)	22 (78.5)	0.470
House dust mite	9 (34.6)	11 (39.2)	0.783
Asthma	0 (0)	0 (0)	—
Atopic Dermatitis	0 (0)	1 (3.5)	1.000
Food allergy	0 (0)	2 (7.1)	0.491
Scores (SE) of pre-pollen season			
Symptom-medication score	0.2 (0.1)	0.4 (0.1)	0.225
Total symptom score	1.0 (0.3)	1.8 (0.5)	0.181
Medication score	0.0 (0.0)	0.1 (0.1)	0.447

Results of the intention-to-treat (ITT) group in percentages, ranges or means ± SDs or SEs. Total symptom score includes total nasal symptom score (runny nose, sneezing, itchy nose, and nasal congestion) and total ocular symptom score (watery eyes and itchy eyes). Total symptom score = total of 6 individual symptom scores, each assessed on a 5-point scale (from 0 = absent to 4 = very severe). Symptom-medication score = average total symptom score (total symptom score/6) plus medication score. Statistical analysis was performed using unpaired *t*-test or Fisher’s exact probability test. M, male; F, female.

**Table 2:** Treatment-emergent adverse events (TEAEs) during oral immunotherapy (OIT).

TEAE, No. (%)	Treat (N = 26)			Placebo (N = 28)		
	Total	Build-up phase	Maintenance phase	Total	Build-up phase	Maintenance phase
Total	6 (23.0)	5 (19.2)	2 (7.6)	5 (17.8)	4 (14.2)	2 (7.1)
Mild (Grade 1)	6 (23.0)	5 (19.2)	2 (7.6)	5 (17.8)	4 (14.2)	2 (7.1)
Moderate (Grade 2)	0 (0)	0 (0)	0 (0)	0 (0)	0 (0)	0 (0)
Severe (Grade 3)	0 (0)	0 (0)	0 (0)	0 (0)	0 (0)	0 (0)
Discontinued immunotherapy	0 (0)	0 (0)	0 (0)	0 (0)	0 (0)	0 (0)
Preferred term, No. (%)						
Oral dysesthesia	0 (0)	0 (0)	0 (0)	1 (3.5)	1 (3.5)	0 (0)
Diarrhea	1 (3.8)	1 (3.8)	0 (0)	0 (0)	0 (0)	0 (0)
Loose stools	0 (0)	0 (0)	0 (0)	1 (3.5)	1 (3.5)	0 (0)
Abdominal pain	1 (3.8)	1 (3.8)	0 (0)	2 (7.1)	0 (0)	2 (7.1)
Abdominal discomfort	1 (3.8)	1 (3.8)	0 (0)	0 (0)	0 (0)	0 (0)
Bloating	1 (3.8)	1 (3.8)	0 (0)	0 (0)	0 (0)	0 (0)
Stomach pain	2 (7.6)	1 (3.8)	2 (7.6)	0 (0)	0 (0)	0 (0)
Chest discomfort	0 (0)	0 (0)	0 (0)	1 (3.5)	1 (3.5)	0 (0)
Laryngopharyngeal dysesthesia	0 (0)	0 (0)	0 (0)	1 (3.5)	1 (3.5)	0 (0)
Rhinorrhoea	1 (3.8)	1 (3.8)	0 (0)	0 (0)	0 (0)	0 (0)
Watering eyes	0 (0)	0 (0)	0 (0)	1 (3.5)	1 (3.5)	0 (0)

AEs were graded according to Co mmon Terminology Criteria for Adverse Event (CTCAE) v4.0/MedDRA v12.0.

**Table 3:** Individual treatment-emergent adverse events (TEAEs) during oral immunotherapy (OIT).

	No.	M/F	AEs	Severity (grade)	Days of OIT	Duration (days)
OIT group	1	M	Rhinorrhea	Grade 1	0	1
	2	F	Diarrhea	Grade 1	5	1
	3	F	Abdominal pain	Grade 1	0	0.5
			Abdominal pain	Grade 1	2	3
	4	F	Stomach pain	Grade 1	0	3
			Bloating	Grade 1	6	1
			Stomach pain	Grade 1	41	1
	5	F	Abdominal discomfort	Grade 1	0	26
	6	F	Stomach pain	Grade 1	25	2
Placebo	1	F	Watering eyes	Grade 1	0	1
	2	F	Oral dysesthesia	Grade 1	13	6
			Abdominal pain	Grade 1	34	6
	3	F	Chest discomfort	Grade 1	0	0.1
			Laryngopharyngeal dysesthesia	Grade 1	0	0.04
	4	F	Loose stools	Grade 1	17	46
	5	F	Abdominal pain	Grade 1	19	3

AEs were graded according to Common Terminology Criteria for Adverse Event (CTCAE) v4.0/MedDRA v12.0. M, male; F, female.

**Table 4:** Adjusted scores during Japanese cedar/cypress pollen season (entire pollen season) and Japanese cedar pollen (JCP) season.

	Active group	Placebo group	Difference	*p* value	95% CI of the difference
	(n = 26)	(n = 28)			
Entire pollen season					
Symptom-medication score					
Mean (SE)^†^	1.44 (0.3)	2.01 (0.3)	−0.56	0.085	−1.21 to 0.08
Median (Q1-Q3)	1.52 (0.89–2.12)	1.91 (1.03–3.01)			
Total Symptom score					
Mean (SE)^†^	6.41 (0.8)	6.28 (0.7)	0.13	0.891	−1.77 to 2.03
Median (Q1-Q3)	5.96 (3.92–8.92)	6.04 (3.88–9.00)			
Medication score					
Mean (SE)^†^	0.41 (0.2)	0.96 (0.2)	−0.54	0.040	−1.06 to −0.02
Median (Q1-Q3)	0.40 (0.09–0.84)	0.55 (0.27–1.54)			
Total nasal score					
Mean (SE)^†^	4.69 (0.6)	4.40 (0.5)	0.28	0.674	−1.07 to 1.64
Median (Q1-Q3)	4.31 (2.92–5.69)	4.96 (2.46–6.35)			
Total ocular score					
Mean (SE)^†^	1.73(0.3)	1.85(0.3)	−0.13	0.703	−0.80 to 0.55
Median (Q1-Q3)	1.69(0.85–2.85)	1.65(1.00–2.81)			
JCP season					
Symptom-medication score					
Mean (SE)^†^	1.69 (0.3)	2.36 (0.3)	−0.66	0.075	−1.40 to 0.07
Median (Q1-Q3)	2.05 (0.99–2.55)	2.21 (1.25–3.45)			
Total Symptom score					
Mean (SE)^†^	7.25 (1.0)	7.13 (0.8)	0.12	0.911	−2.02 to 2.25
Median (Q1-Q3)	7.25 (4.63–10.75)	7.50 (4.38–10.38)			
Medication score					
Mean (SE)^†^	0.51 (0.3)	1.16 (0.2)	−0.65	0.033	−1.24 to −0.05
Median (Q1-Q3)	0.43 (0.07–1.14)	0.79 (0.21–1.73)			
Total nasal score					
Mean (SE)^†^	5.24 (0.7)	4.74 (0.6)	0.5	0.506	−0.99 to 1.99
Median (Q1-Q3)	5.13 (3.50–6.88)	5.31 (2.69–7.13)			
Total ocular score					
Mean (SE)^†^	2.09(0.3)	2.33(0.3)	−0.24	0.538	−1.02 to 0.54
Median (Q1-Q3)	2.13(1.13–3.38)	2.13(1.44–3.31)			

Maximum symptom-medication score (total symptom score/6 plus medication score) = 13; maximum total symptom score = 24; maximum medication score = 9; maximum total nasal score = 16; and maximum total ocular score = 8.

^†^Scores are shown as the least square (LS) means adjusted by analysis of covariance (ANCOVA) model, with symptom-medication score in the pre-pollen season, and sensitization status of cypress pollen and house dust mite as factors in the analysis. Statistical analysis was performed using ANCOVA.
