# Supplementary material for: Safety and efficacy of short-term oral immunotherapy with Cry j 1-galactomannan conjugate for Japanese cedar pollinosis: a randomized controlled trial
Source: Sci Rep. 2017 Apr 11;7:46142. doi: 10.1038/srep46142 (PMC5387405; doi:10.1038/srep46142)
Supplement: Supplementary Information [file srep46142-s1.pdf]

Title

Safety and efficacy of short-term oral immunotherapy with Cry j 1-galactomannan conjugate for Japanese cedar pollinosis: a randomized controlled trial

Authors

Daisuke Murakami, M.D., Ph.D. [a,d,*], Motohiro Sawatsubashi, M.D., Ph.D. [a], Hirofumi Omori , M.D.[a], Akira Saito, Ph.D. [b], Akio Kato, Ph.D. [c], Shizuo Komune, M.D., Ph.D. [a] and Takashi Nakagawa, M.D., Ph.D. [a]

[a] *Department of Otorhinolaryngology, Graduate School of Medical Sciences, Kyushu University, Fukuoka, Japan*

[b] *Bio & Healthcare Business Group, Bio & Healthcare Business Division, Wako Filter Technology Co., Ltd., Ibaraki, Japan*

[c] *Department of Biological Chemistry, Yamaguchi University, Yamaguchi, Japan*

[d] *Department of Otorhinolaryngology, Saiseikai Fukuoka General Hospital, Fukuoka, Japan*

*Corresponding author

Daisuke Murakami, M.D., Ph.D.

Department of Otorhinolaryngology, Graduate School of Medical Sciences, Kyushu University, Maidashi 3-1-1 Higashi-ku, Fukuoka 812-8582, Japan

E-mail: muradai@qent.med.kyushu-u.ac.jp

Supplementary Figure 1: Total symptom score (a) and medication score (b) during pollen season and daily Japanese cedar/cypress pollen counts.

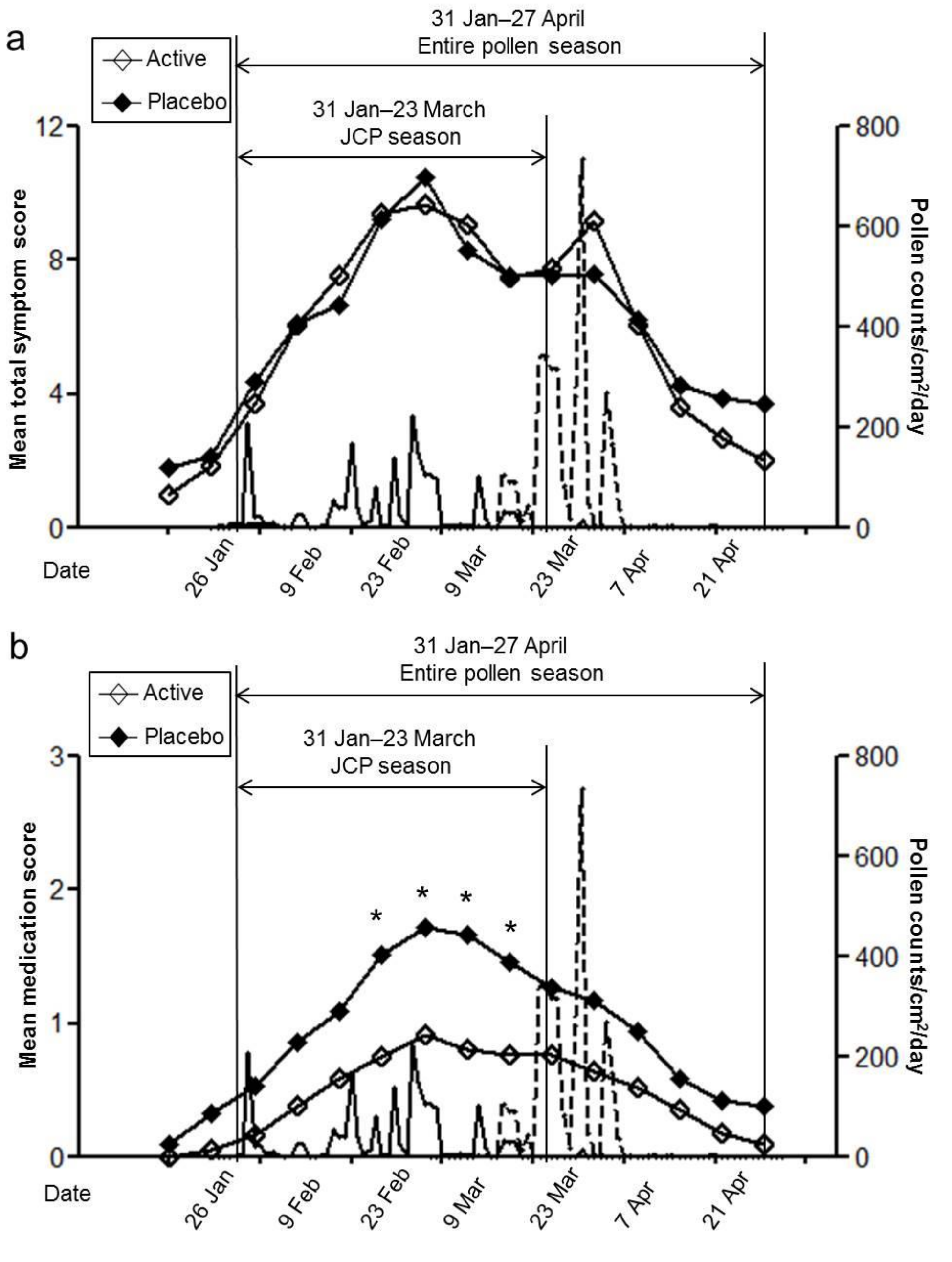

Solid line: Japanese cedar pollen counts. Broken line: Japanese cypress pollen counts. Open squares: mean scores in the active group (n = 26). Solid squares: mean scores in the placebo group (n = 28). * $p < 0.05$, between active and placebo groups. Statistical analysis was performed using unpaired *t*-test.

Supplementary Figure 2: Change in overall symptoms during this pollen season compared with those in the previous pollen seasons.

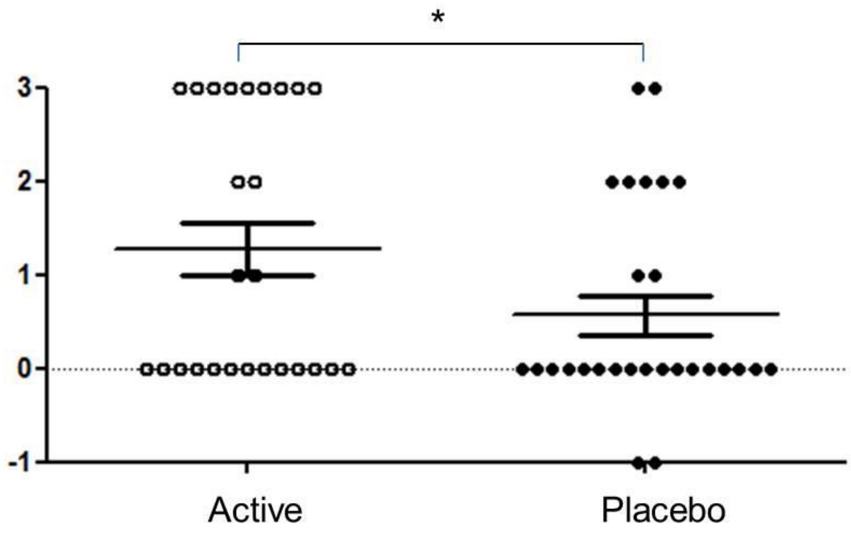

A simple questionnaire (worse = −1, no change = 0, slight improvement = 1, moderate improvement = 2, and marked improvement = 3) about the overall symptoms during the entire pollen season compared with those in the previous years was carried out at the end of this pollen season. Open circles: the active group (n = 26). Solid circles: the placebo group (n = 28). *$p < 0.05$, between active and placebo groups. The bars indicate the means ± SEs. Statistical analysis was performed using an unpaired *t*-test.

Supplementary Table 1: Adjusted symptom score during Japanese cedar/cypress pollen season (entire pollen season) and Japanese cedar pollen (JCP) season.

	Active group (n = 26)	Placebo group (n = 28)	Difference	*p* value	95% CI of the difference
Entire pollen season					
Sneezing					
Mean (SE)†	1.21 (0.15)	1.19 (0.13)	0.02	0.905	−0.32 to 0.36
Median (Q1-Q3)	1.08 (0.85–1.54)	1.31 (0.88–1.65)			
Runny nose					
Mean (SE)†	1.33 (0.18)	1.28 (0.15)	0.05	0.812	−0.35 to 0.44
Median (Q1-Q3)	1.23 (0.85–1.54)	1.42 (0.85–1.73)			
Nasal congestion					
Mean (SE)†	1.12 (0.14)	0.91 (0.13)	0.21	0.208	−0.12 to 0.54
Median (Q1-Q3)	1.00 (0.62–1.46)	1.04 (0.46–1.62)			
Itchy nose					
Mean (SE)†	1.02 (0.17)	1.01 (0.15)	0.01	0.955	−0.37 to 0.39
Median (Q1-Q3)	1.04 (0.54–1.46)	1.04 (0.31–1.62)			
Itchy eyes					
Mean (SE)†	1.06 (0.16)	1.18 (0.14)	−0.12	0.492	−0.49 to 0.24
Median (Q1-Q3)	1.08 (0.69–1.62)	1.15 (0.77–1.58)			
Watery eyes					
Mean (SE)†	0.67 (0.16)	0.66 (0.14)	0.01	0.961	−0.34 to 0.36
Median (Q1-Q3)	0.58 (0.15–1.08)	0.42 (0.15–1.35)			
JCP season					
Sneezing					
Mean (SE)†	1.34 (0.16)	1.29 (0.14)	0.05	0.785	−0.31 to 0.41
Median (Q1-Q3)	1.38 (1.13–1.88)	1.25 (0.94–1.75)			
Runny nose					
Mean (SE)†	1.53 (0.20)	1.38 (0.17)	0.15	0.497	−0.29 to 0.60
Median (Q1-Q3)	1.50 (1.00–1.88)	1.31 (0.75–2.06)			
Nasal congestion					
Mean (SE)†	1.22 (0.16)	0.94 (0.14)	0.28	0.133	−0.09 to 0.64
Median (Q1-Q3)	1.19 (0.63–1.75)	1.19 (0.50–1.69)			
Itchy nose					
Mean (SE)†	1.17 (0.20)	1.11 (0.17)	0.06	0.769	−0.37 to 0.50
Median (Q1-Q3)	1.31 (0.75–1.75)	1.13 (0.50–1.75)			
Itchy eyes					
Mean (SE)†	1.31 (0.18)	1.50 (0.16)	−0.19	0.357	−0.61 to 0.22
Median (Q1-Q3)	1.38 (1.00–1.75)	1.50 (1.00–1.94)			
Watery eyes					
Mean (SE)†	0.79 (0.18)	0.83 (0.16)	−0.04	0.843	−0.45 to 0.37
Median (Q1-Q3)	0.75 (0.13–1.50)	0.69 (0.25–1.25)			

Maximum each symptom score = 4. †Scores are shown as the least square (LS) means adjusted by analysis of covariance (ANCOVA) model, with symptom-medication score in the pre-pollen season, and sensitization status of cypress pollen and house dust mite as factors in the analysis. Statistical analysis was performed using ANCOVA.

Supplementary Table 2: Adjusted scores using ANCOVA including the score at each time point in each patient during Japanese cedar/cypress pollen season (entire pollen season) and Japanese cedar pollen (JCP) season.

	Active group (n=26)	Placebo group (n=28)	Difference	*p* value	95% CI of the difference
Entire pollen season					
Symptom-medication score					
Mean (SE)†	1.44 (0.09)	2.00 (0.08)	−0.565	<0.001	−0.78 to −0.35
Total symptom score					
Mean (SE)†	6.30 (0.32)	6.27 (0.28)	0.033	0.928	−0.68 to 0.74
Medication score					
Mean (SE)†	0.39 (0.07)	0.96 (0.06)	−0.57	<0.001	−0.73 to −0.40
Total nasal score					
Mean (SE)†	4.54 (0.23)	4.45 (0.20)	0.09	0.734	−0.43 to 0.61
Total ocular score					
Mean (SE)†	1.76 (0.11)	1.81 (0.10)	−0.058	0.654	−0.31 to 0.19
JCP season					
Symptom-medication score					
Mean (SE)†	1.56 (0.16)	2.19 (0.14)	−0.638	0.001	−0.99 to -0.28
Total symptom score					
Mean (SE)†	6.77 (0.53)	6.84 (0.47)	−0.067	0.911	−1.25 to 1.11
Medication score					
Mean (SE)†	0.43 (0.12)	1.05 (0.10)	−0.627	<0.001	−0.90 to −0.35
Total nasal score					
Mean (SE)†	4.82 (0.38)	4.57 (0.33)	0.251	0.56	−0.59 to 1.09
Total ocular score					
Mean (SE)†	1.94 (0.19)	2.26 (0.17)	−0.318	0.149	−0.75 to 0.11

Maximum symptom-medication score (total symptom score/6 plus medication score) = 13; maximum total symptom score = 24; maximum medication score = 9; maximum total nasal score = 16; and maximum total ocular score = 8. †Scores are shown as the least square (LS) means adjusted by analysis of covariance (ANCOVA) model, with symptom-medication score in the pre-pollen season, sensitization status of cypress pollen and house dust mite, and the score at each time point in each patient during the entire pollen season and JCP season as factors in the analysis. Statistical analysis was performed using ANCOVA.
